# Development of High-Sensitivity Electrically Conductive Composite Elements by Press Molding of Polymer and Carbon Nanofibers

**DOI:** 10.3390/mi13020170

**Published:** 2022-01-23

**Authors:** Shunsuke Aikawa, Yugang Zhao, Jiwang Yan

**Affiliations:** 1 Department of Mechanical Engineering, Faculty of Science and Technology, Keio University, Hiyoshi 3-14-1, Kohoku-ku, Yokohama 223-8522, Japan; 2 School of Mechanical Engineering, Shandong University of Technology, Zibo 255000, China

**Keywords:** carbon nanofibers, polymer, press molding, laser-induced periodic surface structure, conductive composite element, strain sensor

## Abstract

Carbon nanofibers (CNFs) have various excellent properties, such as high tensile strength, electric conductivity and current density resistance, and thus have great application potential in electrical sensor development. In this research, electrically conductive composite elements using CNFs sandwiched by thermoplastic olefin (TPO) substrates were developed by press molding. The metal mold used for press molding was processed by a femtosecond laser to generate laser-induced periodic surface structures (LIPSS) on the mold surface. The aggregate of CNFs was then flexibly fixed by the LIPSSs imprinted on the TPO substrate surface to produce a wavy conductive path of CNFs. The developed composite elements exhibited a sharp increase in electrical resistance as strain increased. A high gauge factor of over 47 was achieved, which demonstrates high sensitivity against strain when the composite element is used as a strain gauge. Scanning electron microscope observation revealed that the TPO filled the spaces in the aggregate of CNFs after press molding, and the conductive path was extended by the tensile strain. The strain-induced dynamic changes of contact states of CNFs and CNFs networks are discussed based on the electrical performance measurement and cross-sectional observation of the elements. This research provides a new approach to the production of flexible and high sensitivity strain sensors.

## 1. Introduction

Carbon nanofibers (CNFs) are fibrous carbon with a diameter of several tens of nanometers [1,2,3]. Due to their excellent physical properties, such as light specific gravity, high strength and electrical current conductivity, CNFs are in high demand for various fields of industry [4,5,6]. In particular, CNFs are attractive for developing superior conductive elements. Currently, in the field of electronic devices such as strain gauges, damage of the conductive parts caused by excessively high electrical current density and/or large strain in miniaturized devices is a severe problem [7,8,9]. This problem can be solved by applying CNFs in the conductive parts because CNFs possess high electrical current density resistance, which is around one thousand times higher than that of copper [10,11,12] and prevents conductor breakage. This is because the atomic arrangement of CNF in a laminated structure prevents electron scattering and enables high-speed electron movement [13,14,15]. In addition, a CNF aggregate with a network structure is highly flexible, thus able to withstand an extremely large strain. Therefore, CNFs are expected to be a new conductive material for flexible electronic devices that are used in robotics, wearable devices, car manufacturing, and so on.

In recent years, polymer composite materials containing nanocarbons such as CNFs or carbon nanotubes (CNTs) have been developed in various areas [16,17,18]. Kumar et al. reported that by dispersing CNTs in silicone rubber, the composite exhibited accurate resistance response under tensile strain [19]. Yang et al. reported that by dispersing CNTs and graphene in silicone rubber, the composite had high durability and stability of electrical resistance response during cyclic loading [20]. However, in this previous research, CNTs were mixed with silicone rubber with exposed CNTs outside the composite, which is harmful and dangerous for human bodies [21,22]. Furthermore, the reported gauge factors indicating the sensitivity of the sensors was less than 20, which is lower than the gauge factor required for specific applications such as human motion detection (30~) [23,24]. The reason for the limit of the gauge factor is that most CNFs are not directly connected to each other but separated by the polymer matrix, thus conductive networks cannot be effectively generated. In such a case, the decrease in the contacts among CNFs under tension is small, thus the sensitivity is low.

In this research, sandwich-structure composite elements containing CNFs will be fabricated by press molding. In the elements, CNFs are sandwiched by base materials with periodic wavy surface, which is formed by transferring laser-induced periodic surface structure (LIPSS) on the mold, to fix the CNFs. This method has several advantages. First, CNFs are completely sealed between the base materials and exposure of CNFs can be prevented. Second, the wavy conductive path of CNFs expands due to tensile force compared to the linear shape to maintain the length of the conductive path, so it is expected to prevent the conductive path from breaking under tension [25]. Third, this composite structure can reduce the amount of CNFs to less than 1 phr by forming only one layer without dispersing CNFs throughout the polymer; while in previous studies, CNFs more than 3 phr were used [19,20]. Fourth, under tension, a sharp decrease in the contacts among CNFs and an increase in the electrical resistance are achievable; thus, an increase in the sensitivity can be obtained [26,27]. Fifth, the process of press molding can reduce the fabrication time, which is considerably shorter compared with previous research [19,20].

After the fabrication tests, the conductivity of the fabricated elements will be evaluated under external forces. In addition, a model for the contact states of CNFs will be established to describe the CNF networks in the composite elements affecting the resistance response, and the model will be verified by cross-sectional observation of the elements. This study will contribute to the development of low-cost superior conductive elements, which may be applied to high-flexibility and high-sensitivity strain sensors in the future.

## 2. Materials and Methods

### 2.1. Structure Design for Composite Element

**Figure 1:**
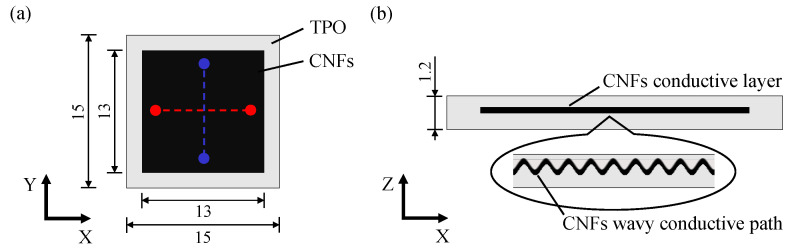
Schematic of the designed TPO-CNFs composite element having a sandwich structure with a nanoscale periodical wavy conductive path: (**a**) top view (unit of length: mm), (**b**) cross-sectional view. The red and blue dots in (**a**) indicate two copper wires inserted into the composite element and connected to CNFs between the two points during measurement of electrical resistance as described in Section 2.5.

The designed structure of the composite element is shown in Figure 1. CNFs are sandwiched by two base materials of polymer with periodic wavy surfaces, which are formed by transferring the LIPSS from the mold surface through press molding. In this structure, CNFs are completely sealed by the polymer base materials to prevent their exposure, and the wavy conductive path of the CNFs expands due to tensile force to prevent the conductive path from breaking.

### 2.2. Materials

As the base material sandwiching CNFs, thermoplastic olefin (TPO) was used in this study. TPO is a mixture of polypropylene (PP) and ethylene-propylene-diene rubber (EPDM), and is highly elastic as compared with pure PP. In TPO, PP acts as a hard segment crosslinking EPDM which is a soft segment, and TPO can be reshaped by high temperature over the softening point of PP. By using the TPO substrates, low-cost conductive elements with high flexibility can be developed, which is useful for strain sensor applications in robotics and wearable devices, etc. TPO substrates (MISUMI Group Inc., Tokyo, Japan) with 1.0 mm thickness were used, the physical properties of which are shown in Table 1. The substrates were cut into 15 mm squares for bases and covers. Subsequently, a nanoscale periodic wavy structure was transferred from an aluminum mold to the surface of the TPO substrates by press molding. The LIPSS on the aluminum mold was generated by femtosecond laser irradiation. The irradiation conditions are shown in Table 2. 

The physical properties of CNFs (Mitsubishi Materials Corp., Tokyo, Japan) used in the experiments are shown in Table 3. CNFs were mixed with absolute ethanol at a ratio of 1 mg CNFs: 1 mL ethanol, and the mixture was applied onto the TPO surface with LIPSS. The samples were cleaned by ultrasonic cleaning for 10 min, after which the ethanol was blown off using an air duster. After that, the flat TPO substrate with 1.0 mm thickness was placed on it.

### 2.3. Press Molding for Composite Structure Fabrication

**Figure 2:**
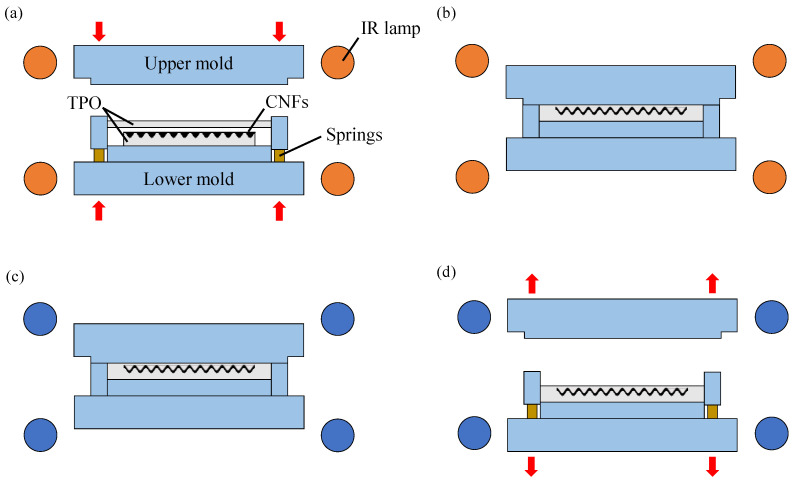
Process flow of the second molding press for sandwiching CNFs with TPO plates: (**a**) heating, (**b**) pressing, (**c**) cooling, (**d**) demolding.

The sandwich structure containing CNFs was fabricated by second press molding as illustrated in Figure 2. The lower mold was raised up towards the upper mold and the mold set was heated by surrounding circular infrared lamps. Subsequently, the two molds were closed, and compression was started after a specific temperature was reached. As a result, the substrates on both sides were combined and CNFs were sealed inside the element. After that, the molds were cooled and opened. Finally, a sandwich structure as illustrated in Figure 2d was obtained. The press molding experiment was performed by using a high-precision molding machine, GMP211 (Toshiba Machine Co. Ltd., Tokyo, Japan). This machine can raise the heating temperature to 800 °C with an accuracy of ±1 °C, and apply a pressing force in the range of 0.2 kN to 20 kN with a resolution of 0.98 N. To prevent the sample and mold from oxidation, press molding was performed in argon gas atmosphere.

The molding conditions are shown in Table 4. As the softening point of PP ranges from 160 °C to 168 °C, in this experiment, the heating temperature was set to 160 °C so that only the surface layers of the TPO softened. As a result, the upper TPO substrate wrapped and adhered to the lower TPO substrate with a periodic structure where CNFs were applied.

In this study, three composite elements under the conditions shown in Table 5 were prepared for comparative analysis. First, to investigate the effect of second molding for forming a sandwich structure, two types of samples with and without the second molding were prepared. In addition, to examine the effect of periodic structure, a sample where CNFs are applied on the original TPO surface without the periodic structure was also prepared. By comparing the results in the three elements, effects of periodic structure and second molding for a sandwich structure were analyzed.

### 2.4. Investigation of CNFs Network Formation

The states of CNFs in the element before and after press molding were observed and compared by using field emission scanning electron microscope (FE-SEM). In addition, changes in the state of the wavy conductive path formed by the CNF aggregate under tensile strain were also observed. Based on the observations, a model for describing the contact states of CNFs and the conductive properties of the composite elements was established for discussion.

### 2.5. Conductivity Evaluation

Electrical resistance of the fabricated composite element was measured by the four-terminal method using a DC power supply device (PAS 160-2, Kikusui Electronics Corporation, Yokohama, Japan) and two digital multi-meters (TY530, Yokogawa Test & Measurement Corporation, Tokyo, Japan). The voltage was set to 10 V. In this measurement, two copper wires were inserted into the composite element and connected to CNFs between the two points (red/blue dots in Figure 1a) in each direction. The electrical resistance was calculated from the electrical current and voltage measured by each digital multi-meter. In order to investigate the difference in conductivity depending on the tensile direction, the resistance was measured under the tensile strain in each of the X-axis and Y-axis directions set as shown in Figure 1.

In the experiment, the change in the electrical resistance with strains was examined. One side of the element was fixed with a vise. Following this, weights in a range of 0–500 g were attached at the opposite side to generate tensile strains in the conductive part. The strain was calculated by the following equation:

(1) $$\varepsilon =\frac{\sigma }{E}=\frac{mg}{Ewt}$$

where *ε* is strain, *σ* is tensile stress, *E* is tensile modulus of TPO, m is weight mass, *g* is gravitational acceleration, *w* is cross-sectional width and *t* is cross-sectional thickness. In the case of the periodic structure with LIPSS transferred, surface electrical resistance was used instead of electrical resistance. The surface electrical resistance was used when a conductive part is a thin film. The surface resistance generally follows:

(2) $$R_{s}{=\rho }_{s}\frac{l}{kw}$$

where *R*_s_ is the surface resistance, *ρ*_s_ is electrical resistivity, *l* is the distance between two points to be measured and *kw* indicates width of conductive area due to CNF aggregate. Therefore, by substituting *w* in Equation (1) to Equation (2), the electrical resistance *R*_s_ should be proportional to the strain *ε* as the following equation:

(3) $$R_{s}=\frac{{E\rho }_{s}tl}{kmg}\varepsilon$$

In addition, the gauge factor of the fabricated composite element, which is also an important coefficient representing the sensitivity of strain gauges, was calculated. The gauge factor was calculated by the following equation:

(4) $$K=\frac{1}{\varepsilon }\frac{\Delta R}{R_{0}}\;$$

where *K* is the gauge factor, Δ*R* is the electrical resistance change to strain, and *R*_0_ is initial electrical resistance. In this study, the conductivity of composite elements was evaluated in terms of increased electrical resistance to strain and gauge factor. Following this, the effect of the periodic structure and pressing force in the fabrication process were investigated.

## 3. Results and Discussion

### 3.1. Wavy Conductive Path Formation Process

**Figure 3:**
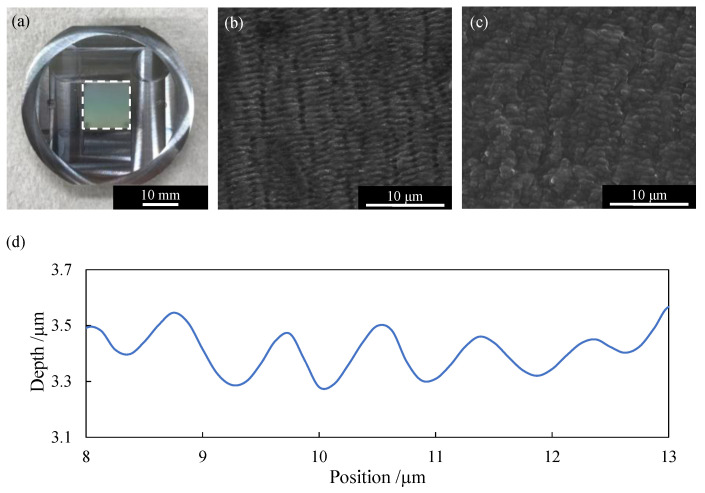
(**a**) An aluminum mold with LIPSS on the region surrounded by the white dotted line; (**b**) FE-SEM micrograph of the LIPSS on the mold surface; (**c**) FE-SEM micrograph of the replicated periodic surface structure on TPO; (**d**) Cross-sectional profile of the periodic structure in (**c**).

In this study, a mold on which LIPSS was formed by laser irradiation was used for the first press molding to generate nanotextures. After that, the sandwich structure of TPO with the periodic structure based on LIPSS was formed by the second press molding. Figure 3a is a photograph of a mold with LIPSS in the square region in the center. The FE-SEM observation result of the LIPSS is shown in Figure 3b. Numerous nanoscale periodic grooves are confirmed. By transferring the LIPSS to TPO by the first press molding, the periodic structure was replicated from the mold onto the TPO surface, as shown in Figure 3c. The surface profile measured by a laser microscope is shown in Figure 3d. The replicated nanotexture has a depth of ~ 400 nm and a width of ~ 800 nm.

**Figure 4:**
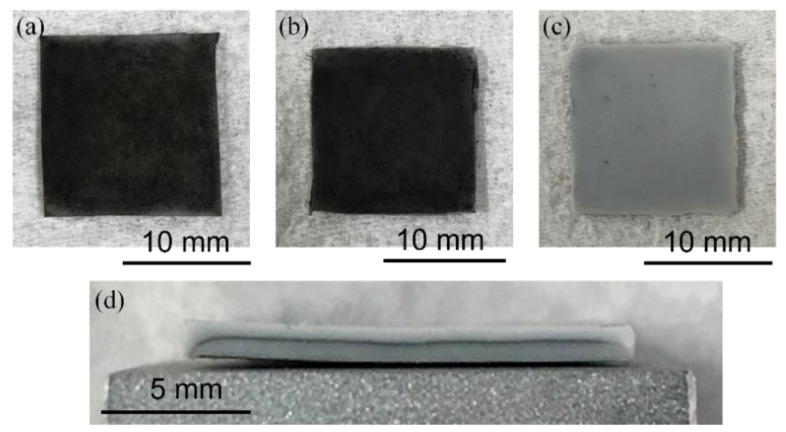
Photographs of fabricated TPO-CNFs composite elements: (**a**) sample A without periodical structure, (**b**) sample B with periodic structure, (**c**) sample C with periodic structure and sandwich structure, (**d**) cross-sectional view of sample C showing a layer of CNFs sandwiched by TPO.

The three types of fabricated composite elements are shown in Figure 4. In Figure 4a,b, the samples A and B look black because the CNFs were exposed to air. The sample C in Figure 4c looks gray due to the CNFs being covered inside the sandwich structure. As shown in Figure 4d, a thin layer of CNFs is uniformly formed inside the sandwich structure (sample C).

**Figure 5:**
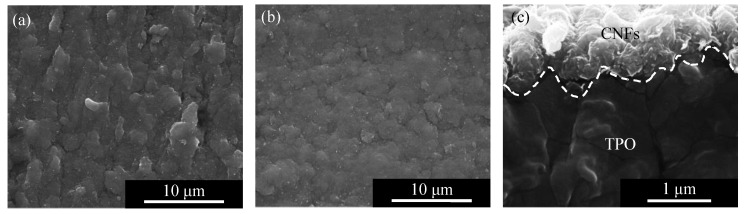
FE-SEM micrograph of CNFs on the TPO surface: (**a**) top view of sample A without periodical structure, (**b**) top view of sample B with periodic structure, (**c**) side view of sample B with periodic structure.

Next, the CNFs on the surfaces of samples A (without LIPSS) and B (with LIPSS) were observed by SEM and the results are shown in Figure 5. The CNFs on the periodic structure of sample B were flatter than those of sample A. This difference might be due to the surface roughness of the substrates. As measured by a laser microscope, the original surface without LIPSS formation (sample A) had a surface roughness of 270 nm Sa, which is rougher than the periodic structure surface (sample B) with a surface roughness of 155 nm Sa. Furthermore, as shown in the cross-sectional view in Figure 5c, the periodic structure on the surface of sample B was filled with CNFs. The CNFs were fixed to the surface even after ultrasonic cleaning. 

**Figure 6:**
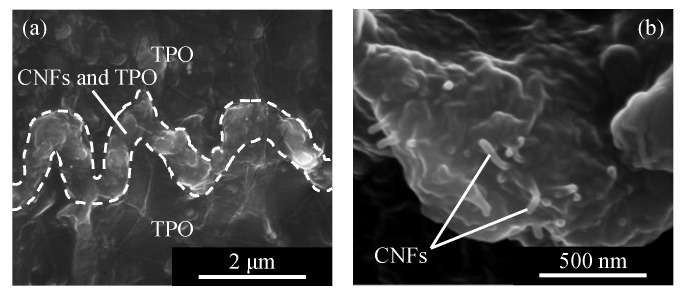
FE-SEM micrograph of the wavy conductive path of CNFs formed in sample C: (**a**) cross-sectional general view, (**b**) enlarged view of the conductive path showing CNFs embedded in the TPO matrix.

The layer of CNFs in the cross section of sample C was observed as shown in Figure 6. There is a wavy path in Figure 6a, which consists of CNFs as shown in the enlarged image Figure 6b. In Figure 6b, it can be seen that the CNFs protruded from the layer due to internal pressure. These facts indicate that CNFs were compressed and deformed according to the surface shape of the periodic structures. In addition, in the conductive wavy layer, CNFs were included in the TPO matrix. It can be presumed that the TPO layer had been softened in the heating process and entered into the CNF aggregate.

**Figure 7:**
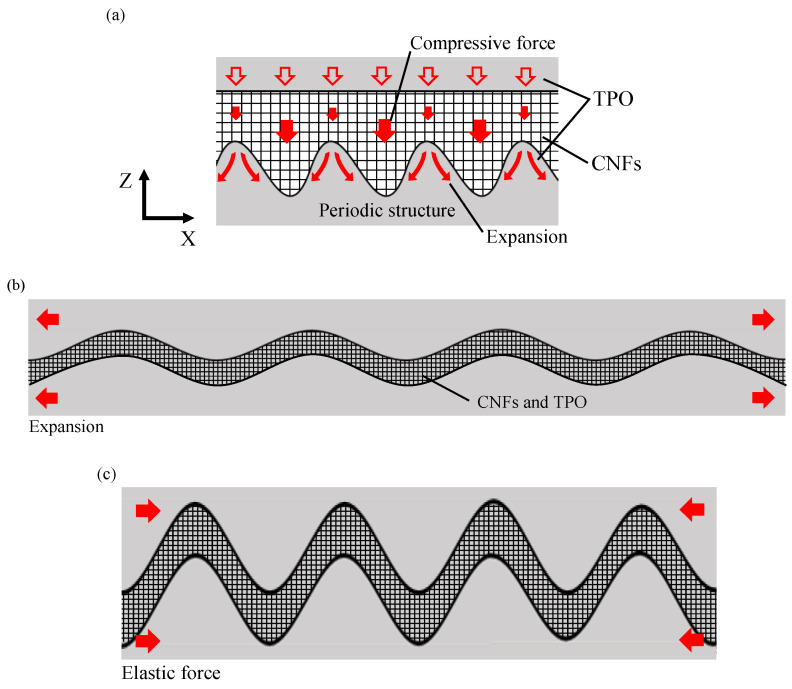
Schematics of formation process of a wavy conductive path: (**a**) before press molding, (**b**) during heating and pressing, (**c**) after press molding.

In Figure 6, it is noted that both the depth and width of the conductive path are approximately 1.6 μm, larger than the depth and width of a single groove in the original periodic structure, i.e., depth ~ 400 nm and width ~ 800 nm. This means that the periodic structure was deformed by the press molding process. A schematic diagram of the formation process of the wavy path of CNFs is illustrated in Figure 7. Before press molding, the periodic structure was filled with CNFs. Following this, the upper TPO substrate was pressed against the CNF aggregate by press molding. At this time, since the surface of TPO was softened by the heating process, the periodic structure of the lower substrate was deformed by the pressing force transmitted from the upper substrate and CNF aggregate. In addition, since the pressing force was applied evenly on the upper and lower planes of TPO substrates, the upper TPO sank significantly above the lower part of the periodic structure. As a result, a wavy conductive path in which the depth of each groove in the periodic structure decreased and the width increased was formed between the upper TPO substrate and lower one by press molding. Due to the heating process at the softening point of TPO, plastic deformation occurred to the TPO to accommodate the interfacial structure. After that, through the cooling process, the extended wavy path was folded in the plane direction. This was due to elastic shrinkage of TPO by releasing compression after the demolding process [25,28,29]. When elastomer materials have a periodic wavy shape during tension, they shrink when released from a tensile force, forming wrinkles with increased depth and decreased width [25,30]. This means that TPO is flexible and has high elasticity. In sample C, the wavy path was folded by the elasticity, but a width larger than the original periodic surface was maintained. As a result, the conductive path shrank and had a periodic shape with increased depth and width.

### 3.2. Behavior of CNFs under Tension

**Figure 8:**
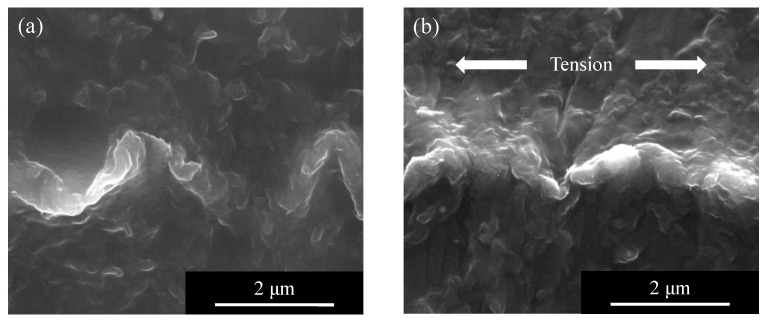
FE-SEM micrograph of the wavy conductive path of CNFs inside TPO: (**a**) without tension, (**b**) under tension.

The cross section of a C type composite element under tensile strain observed from a side direction is shown in Figure 8. The wavy path formed by press molding as shown in Figure 8a was extended by an external tensile force as shown in Figure 8b. The width of the periodic conductive path increased while the depth decreased. 

**Figure 9:**
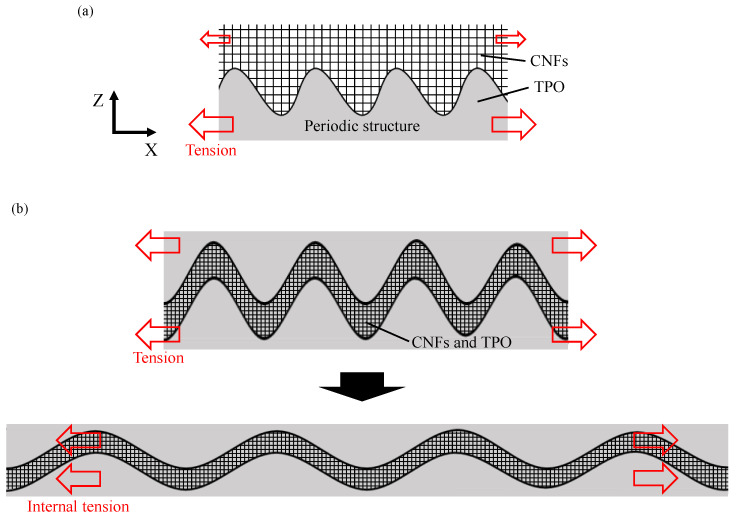
Schematics of deformation behavior of CNF aggregate: (**a**) CNFs on periodic structure before TPO sandwiching, (**b**) deformation of CNF layer in TPO under tension.

The effect of the sandwich structure on the behavior of CNFs under a tensile force applied to the TPO substrate is illustrated in Figure 9. In the case of sample B, the upper fibers in the CNF aggregate did not completely follow the extension of the base TPO because CNFs in the upper layer did not contact the TPO directly, as illustrated in Figure 9a. On the other hand, in the case of sample C, TPO existed around the CNFs as illustrated in Figure 9b. Therefore, the wavy path of the CNFs was easily deformed by forces from the surrounding TPO [25,28,29]. Due to the sensitive deformation corresponding to TPO deformation, the fibers changed their position in the wavy path regardless of the upper and lower parts, and the contact state changed [31].

**Figure 10:**
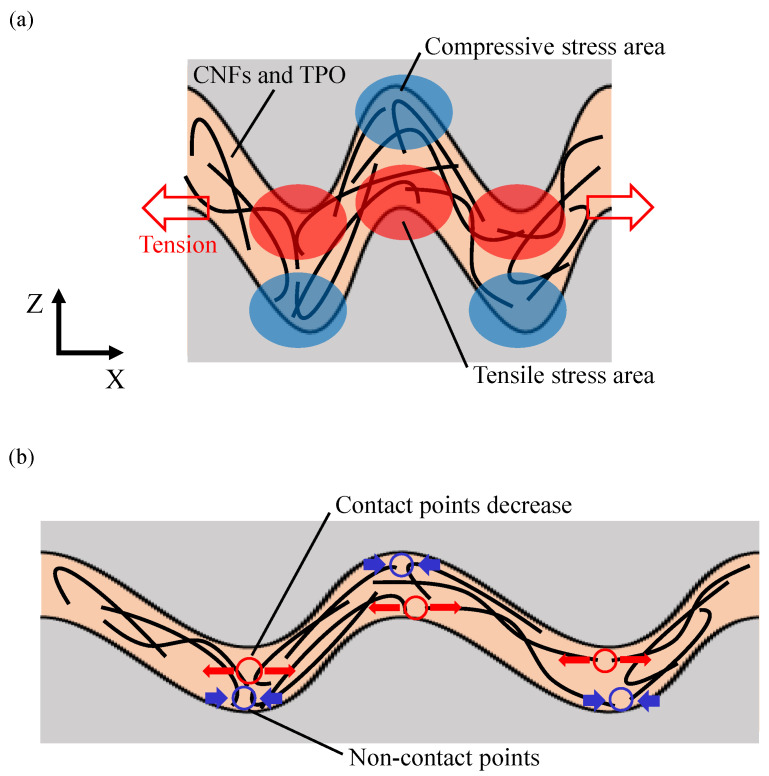
Schematic of contacting behavior of CNFs inside the conductive path: (**a**) without tension, (**b**) under tension.

In addition, diagrams of the microscopic behavior of CNFs in the sandwiched conductive path are illustrated in Figure 10. When the wavy path was expanded under tension, tensile stress and compressive stress were generated depending on the location in the path as shown by the red areas and blue areas in Figure 10a. As a result, the contact points between fibers decreased in the part where tensile stress is generated. However, the contact points did not increase in the part where compressive stress was generated, as illustrated in Figure 10b [32]. This is because TPO, softened during the heating process, enters into the CNF aggregate and prevents an increase in the contact points between fibers. Similarly, the contact points did not increase even with compressive stress generated in the cross section of the path [19,26,27].

### 3.3. Electrical Resistance Evaluation

**Figure 11:**
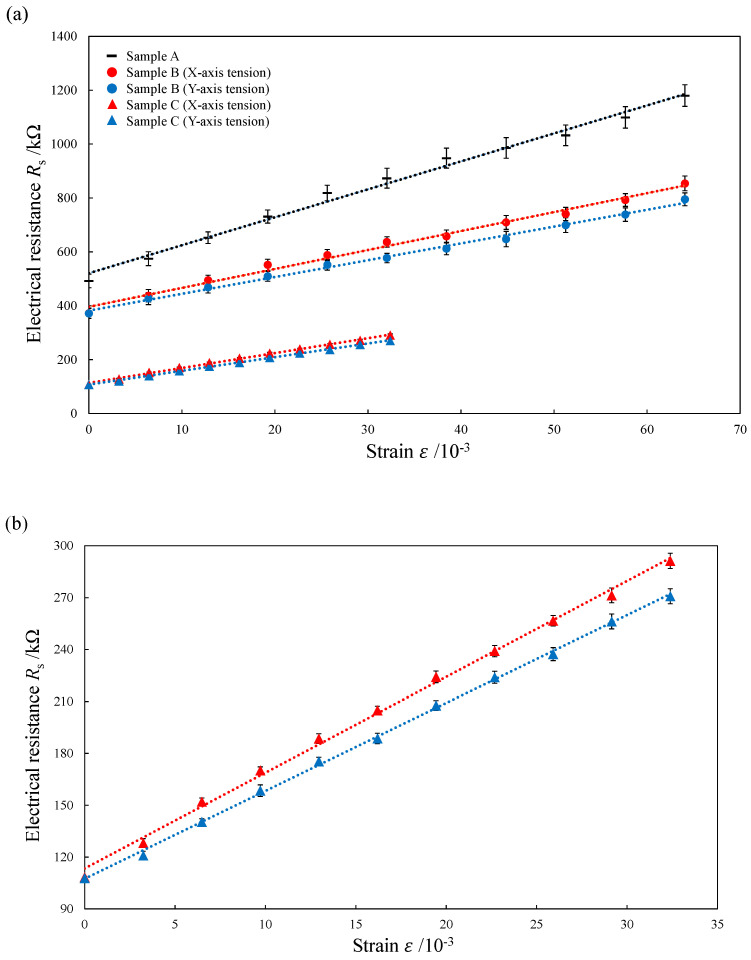
Changes in electrical resistance of TPO-CNF composite elements with different strains: (**a**) samples A, B, and C, (**b**) magnified plot of the result of sample C.

The measured electrical resistance under a varying strain is shown in Figure 11. Two tests for each condition were caried out to confirm the repeatability. The electrical resistance of the composite elements increased in proportion to the increase in tensile strain for all the samples. However, each composite element had a different initial electrical resistance. The electrical resistance of sample B was always smaller than that of sample A, and the initial resistance was reduced by 24.4%. This indicates the effect of the periodic structure surface. There was a uniform conductive path with continuous CNF connection on the periodic structure surface with smaller surface roughness. On the other hand, the conductive path on the flat surface without LIPSS is not uniform due to the irregular CNF aggregates. As a result, the conductive path in which CNFs were evenly distributed on the periodic structure without height difference reduced the electrical resistance. In addition, the electrical resistance of sample C is always the smallest among the three elements. The initial resistance is reduced by 70.9% as compared with sample B as shown in Figure 11a, indicating the effect of the compression by the sandwich structure during the second press molding. The compression increases the density of CNF aggregate and decreases the electrical resistance of the fabricated element [26,27].

### 3.4. Gauge Factor Evaluation

**Figure 12:**
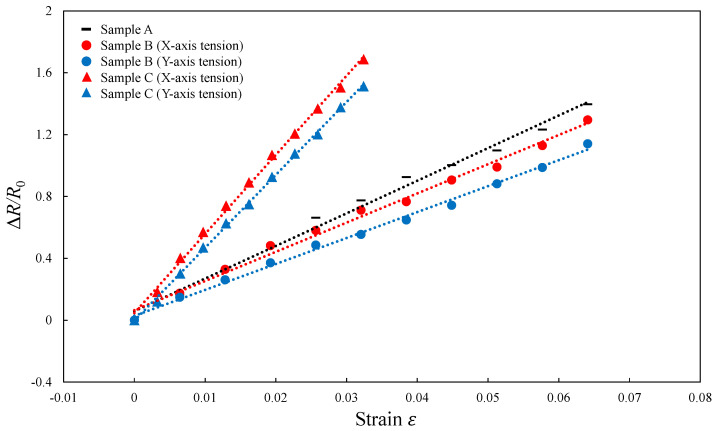
Plots of resistance change at various strains showing the gauge factors of different types of TPO-CNF composite elements.

The gauge factors of composite elements were calculated by Equation (4) and the result is illustrated in Figure 12. The gradient of the lines in the figure indicates the gauge factor. The gauge factor for sample A is 19.9, which is higher than that of X-direction tension (17.7) and Y-direction tension (16.3) for sample B. This is because in sample B, which had a smaller height difference in the conductive path, fiber contacts were maintained in the compression direction during the tension of TPO substrates, and the increase in electrical resistance was suppressed [19,26,27]. In addition, the gauge factor of X-direction tension for sample C is 48.9, and that on Y-direction tension is 47.3, which are distinctly higher than those of other types of conductive elements, as well as the CNF-based conductive elements, which had a gauge factor of 17.5 in a previous study [19,20]. This indicates that the electrical resistance changes sensitively even at a small strain; in other words, the fabricated elements are highly sensitive. 

The high sensitivity of sample C is due to the wavy conductive path of the CNFs. As shown in Figure 9b, the wavy conductive path extends under tension, but the path length does not change. The high sensitivity, due to the increased electrical resistance even though the length of the conductive path did not change, indicates that the contact state of fibers inside the CNF aggregate was changed. Sample C exhibited the highest sensitivity because the fiber contact was decreased more than the other composite elements under the same tensile stress. This is because TPO prevents the increase in contacts between fibers in the compressed parts inside the wavy conductive path, as shown in Figure 10. As a result, suppression of the increase in the electrical resistance due to the compressive force does not occur. Therefore, the sensitivity of the composite element with wavy conductive path increased due to the significant decrease in the contact points of CNFs.

In the case of tension in the Y-direction, gauge factors became smaller in both sample B and sample C. This is because the compression state for each tension direction was different. In the X-direction tension, the electrical resistance increased as the cross-sectional area of the conductive path was compressed. However, in Y-direction tension, the wavy conductive path was already formed in the cross section. Since the wavy conductive path was folded by compression, the cross-sectional area of the conductive path was not decreased [25,28,29]. Therefore, the increase in the electrical resistance was suppressed in the Y-direction tension and the sensitivity decreased. 

As shown in previous work, carbon nanotubes and conductive polymers exhibit a nonlinear current voltage characteristic [33,34]. In this study, under the measuring conditions used, no clear nonlinearity was found in the results. This might be one of the features of the developed sensor structure. Another possibility is nonlinearity might take place if other measuring conditions are used. This issue will be further investigated in the future during application developments.

It should also be mentioned that the TPO substrate thickness will affect the elastic modulus, and in turn, the performance of the strain sensor. In this study, we used a thick TPO substrate because the sensor we developed had a very high sensitivity which could detect the resistance change even for a very small bending strain. For highly flexible sensors, it may be necessary to use thinner TPO substrates which enable large bending strains. This may lead to different deformation behaviors of CNFs and the final performance of the sensor such as maximum strain. These aspects will be investigated in our future work.

## 4. Conclusions

TPO-CNF composite elements with nanoscale periodic interfacial structures were fabricated by press molding, and their electrical properties were evaluated. The conclusions are as follows:

(1) The surface periodic structure of TPO transferred from a mold with LIPSS exhibited a strong effect of retaining CNFs and unifying the CNF distribution. As a result, the electrical resistance was decreased.
(2) A wavy conductive path of CNFs was formed based on the periodic structure of TPO surface. Due to the compression of the CNF aggregate by a pressing force, the contact points among the fibers increased. The conductive path decreased the electrical resistance by approximately 70% under no tension.
(3) Under tension, the contact points of CNFs decrease in the wavy conductive parts where tensile stress is generated, while in locations where compressive stress is generated, the contact points are less affected.
(4) The composite element with a sandwich structure showed a gauge factor over 47 which is several times higher than that of conventional CNF-based conductive elements reported in previous studies, indicating significantly higher sensitivity as a strain sensor.

This study demonstrated the feasibility of fabricating highly sensitive, compact, flexible and harmless composite conductive elements from CNFs and polymers by press molding. It is expected that this kind of conductive element might be useable as low-cost and high-performance strain sensors in flexible electronic devices, robots, wearable devices, car manufacturing and so on.

## Figures and Tables

**Table 1:** Physical properties of TPO.

Parameter	Value
Density [g/cm^3^]	0.918
Tensile modulus [MPa]	8.41
Tensile strength [MPa]	8.3
Poisson’s ratio	0.419

**Table 2:** Laser irradiation conditions for LIPSS generation.

Parameter	Value
Pulse width [fs]	256
Frequency [kHz]	100
Laser power [mW]	50
Scanning speed [mm/s]	50
Repetition [times]	3
Pitch [µm]	10

**Table 3:** Physical properties of CNFs.

Parameter	Value
Diameter [nm]	0.918
Length [µm]	8.41
Volume resistivity [Ω·cm]	8.3
Specific surface area [m^2^/g]	0.419

**Table 4:** Press molding conditions.

Parameter	Value
Pressing force [kN]	1.0 (1st press for transferring LIPSS) 0.2 (2nd press for closing sandwich structure)
Pressing time [s]	320
Heating and pressing holding time [s]	20
Heating temperature [℃]	160
Cooling temperature [℃]	90

**Table 5:** Press molding steps used for different samples.

	1st Press for Transferring LIPSS	2nd Press for Closing Sandwich Structure
Sample A	No	No
Sample B	Yes	No
Sample C	Yes	Yes
